# Suppression of Ocular Vascular Inflammation through Peptide-Mediated Activation of Angiopoietin-Tie2 Signaling

**DOI:** 10.3390/ijms21145142

**Published:** 2020-07-21

**Authors:** Adam C. Mirando, Raquel Lima e Silva, Zenny Chu, Peter A. Campochiaro, Niranjan B. Pandey, Aleksander S. Popel

**Affiliations:** 1 Department of Biomedical Engineering, Johns Hopkins University School of Medicine, Baltimore, MD 21205, USA; amiran10@jhmi.edu (A.C.M.); zchu1@jhu.edu (Z.C.); 2 Department of Ophthalmology and the Wilmer Eye Institute, Johns Hopkins University School of Medicine, Baltimore, MD 21205, USA; rsilva2@jhmi.edu (R.L.e.S.); pcampo@jhmi.edu (P.A.C.); 3 AsclepiX Therapeutics, Inc., Baltimore, MD 21211, USA

**Keywords:** AXT107, peptide therapeutic, ocular diseases, endothelial activation, vascular permeability, vascular leakage, TNFα, NF-κB, IκBα, leukostasis, VCAM-1

## Abstract

Persistent inflammation is a complication associated with many ocular diseases. Changes in ocular vessels can amplify disease responses and contribute to vision loss by influencing the delivery of leukocytes to the eye, vascular leakage, and perfusion. Here, we report the anti-inflammatory activity for AXT107, a non-RGD, 20-mer αvβ3 and α5β1 integrin-binding peptide that blocks vascular endothelial growth factor (VEGF)-signaling and activates tyrosine kinase with immunoglobulin and EGF-like domains 2 (Tie2) using the normally inhibitory ligand angiopoietin 2 (Ang2). Tumor necrosis factor α (TNFα), a central inflammation mediator, induces Ang2 release from endothelial cells to enhance its stimulation of inflammation and vascular leakage. AXT107 resolves TNFα-induced vascular inflammation in endothelial cells by converting the endogenously released Ang2 into an agonist of Tie2 signaling, thereby disrupting both the synergism between TNFα and Ang2 while also preventing inhibitor of nuclear factor-κB α (IκBα) degradation directly through Tie2 signaling. This recovery of IκBα prevents nuclear factor kappa-light-chain-enhancer of activated B cells (NF-κB) nuclear localization, thereby blocking NF-κB-induced inflammatory responses, including the production of VCAM-1 and ICAM-1, leukostasis, and vascular leakage in cell and mouse models. AXT107 also decreased the levels of pro-inflammatory TNF receptor 1 (TNFR1) without affecting levels of the more protective TNFR2. These data suggest that AXT107 may provide multiple benefits in the treatment of retinal/choroidal and other vascular diseases by suppressing inflammation and promoting vascular stabilization.

## 1. Introduction

Inflammation is an important component and underlying cause of many ocular diseases such as diabetic macular edema (DME), retinal vein occlusion (RVO), diabetic retinopathy (DR), dry and wet age-related macular degeneration (AMD), uveitic glaucoma, macular edema secondary to uveitis, and dry eye disease (DED) [1,2,3,4,5,6]. Diseases like DME and wet AMD are now successfully treated with anti-vascular endothelial growth factor (VEGF) agents, but many patients still do not see improvements of more than 15 letters when treated with these agents. Using an agent that inhibits VEGF signaling, activates tyrosine kinase with immunoglobulin and EGF-like domains 2 (Tie2), and helps resolve underlying inflammation as a monotherapy may improve outcomes for these patients. Highlighting the role of inflammation in ocular diseases, anti-inflammatory agents such as corticosteroids have been successfully used to treat DME, anti-tumor necrosis factor α (TNFα) antibodies have been used to treat uveitis, and other anti-inflammatory agents, such as complement inhibitors, are being developed to treat DED [6,7]. However, chronic use of corticosteroids can cause cataracts and glaucoma while anti-TNFα antibodies can increase patients’ susceptibilities to infectious diseases, cancer, and other complications [8]. Safer alternatives to these agents that can be used chronically with minimal side effects are urgently needed.

In ocular diseases, the vasculature plays a critical role in the inflammatory response as it is the frontline interface between the immune system and the systemic tissues. Immune cells circulate through the vasculature and can exit the vessels at points of inflammation, which are marked by increased levels of cell adhesion receptors including VCAM-1 and ICAM-1. The increased levels of these receptors allow rolling, adhesion, and eventual extravasation of the immune cells into the tissues in which they respond to an existing threat such as an infection. The immune cells also secrete pro-inflammatory cytokines such as TNFα that amplify the inflammatory response [9]. Increased levels of these pro-inflammatory cytokines can cause vascular leakage and, when this process is uncontrolled, can lead to edema and vision impairment or loss.

TNFα is a primary cytokine involved in inflammatory disease of the eye and other organs in the body and it is the primary drug target for treating many inflammatory diseases including those affecting the eye. TNFα exists in a soluble and membrane bound form and is a homotrimeric cytokine. Binding of TNFα to its two receptors TNF receptor 1 (TNFR1) and TNFR2 can result in multiple downstream effects including inflammation, cell proliferation, apoptosis, tissue degeneration, host defense, necroptosis, and even tissue regeneration [10]. Anti-TNFα antibodies are very effective in treating many inflammatory diseases, but they have many side-effects because of the complex and often opposing effects of TNFα binding to its receptors. A greater understanding of the downstream effects of TNFα has suggested that agents that only target TNFR1 without targeting TNFR2 may have fewer side effects [10]. The mechanism by which TNFα causes inflammation has been elucidated in great detail [10]. Very briefly, upon TNFα binding to TNFR1, a large complex of proteins assembles at the receptor and relays information into the cytoplasm that results in the phosphorylation and subsequent proteasome-mediated degradation of inhibitor of nuclear factor-κB (IκB), an inhibitor of the transcription factor nuclear factor kappa-light-chain-enhancer of activated B cells (NF-κB) Once free of its inhibitor, NF-κB, which has been identified as a central regulator of ocular inflammation, enters the nucleus and turns on the transcription of many different pro-inflammatory cytokines [11]. NF-κB also induces the synthesis of its inhibitor IκB once it enters the nucleus, which, in turn, sequesters NF-κB in the cytoplasm and shuts off TNFα-induced signaling and inflammation [12].

Angiopoietin 2 (Ang2) is another mediator of inflammation that can potentiate the activities of inflammatory cytokines, including TNFα. Under inflammatory conditions, Ang2 is rapidly secreted from endothelial cell (EC) Weibel–Palade bodies and antagonizes the vessel-stabilizing activities of the Tie2 receptor by blocking its interaction with its canonical agonistic ligand, Ang1 [13]. The reduction in Tie2 signaling results in the weakening of EC–EC junctions and the subsequent leakage of plasma proteins that promote inflammatory processes through the formation of a supportive provisional matrix for extravasating leukocytes [14]. In severe cases such as sepsis or acute respiratory distress syndrome (ARDS), this leakage can lead to shock or impaired organ function through edema [15,16]. In support of its pro-inflammatory role, increases in the levels of circulating Ang2 are proportional to the severity of their conditions [17,18], while the depletion or knockdown of Ang2 or conversion of Ang2 to a Tie2 agonist by an experimental antibody was found to reduce vascular leakage and improve survival in animal models of sepsis [19,20,21,22]. Ang2 also sensitizes ECs to TNFα, enhancing their expression of immune adhesion molecules and stimulating angiogenesis and vascular remodeling [23,24,25]. Moreover, Tie2 activation appears to directly regulate NF-κB-mediated inflammation through interactions with the A20 binding inhibitor of NF-κB 2 (ABIN2) [26,27].

AXT107 is a non-RGD 20-mer α_v_β_3_ and α_5_β_1_ integrin-binding peptide that has previously been shown to inhibit VEGF signaling and stimulate Tie2 through the conversion of the normally inhibitory Ang2 into an activator [28,29]. When injected into the eye, the effects of AXT107 are sustained over a period of at least two months through the formation of a slow-release gel in the vitreous [28]. Furthermore, intraocular injection of AXT107 strongly suppresses VEGF-induced vascular leakage, but also reduces vascular leakage induced by intraocular injection of lipopolysaccharide (LPS) [29]. In this study, we investigated the mechanism by which AXT107 suppresses the more physiologically relevant TNFα-induced inflammation and vascular leakage. Impressively, AXT107 was found to increase the recovery of endothelial cells from inflammation by activating Tie2 through the conversion of endogenous Ang2 that is released from these cells following TNFα exposure. This increase in Tie2 activation strongly counteracted the pro-inflammatory synergy between TNFα and Ang2, as well as initiated Tie2-mediated anti-inflammatory pathways. These data help to elucidate the role of Ang2 in inflammation and suggest that AXT107 has therapeutic potential for the treatment of ocular diseases complicated by inflammation. To our knowledge, these data also establish AXT107 as the first potential monotherapy of ocular vascular diseases that simultaneously inhibits angiogenesis, vascular permeability, and inflammation.

## 2. Results

### 2.1. AXT107 Inhibits TNFα-Mediated Inflammation

**Figure 1:**
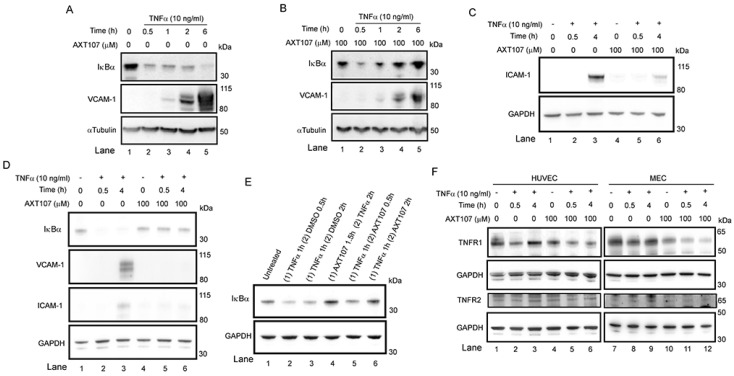
AXT107 regulates tumor necrosis factor α (TNFα) signaling in endothelial cells. (**A**–**D**) Confluent human umbilical vein endothelial cells (HUVECs) (**A**–**C**) or microvascular endothelial cells (MECs) (**D**) pre-treated with 100 µM AXT107 or equivalent DMSO vehicle as indicated for 90 min, followed by varying durations of 10 ng/mL TNFα, and analyzed by Western blot. Targets included inhibitor of nuclear factor-κB (IκBα), VCAM-1, ICAM-1, TNF receptor 1 (TNFR1), and TNFR2. αTubulin or glyceraldehyde 3-phosphate dehydrogenase (GAPDH) is provided as the loading control. (**E**) Confluent HUVECs untreated (lane 1), pretreated with TNFα for 1 h followed by DMSO (lanes 2 and 3) or 100 µM AXT107 (lanes 5 and 6) for 30 min or 2 h, or pretreated with 100 µM AXT107 for 90 min followed by TNFα for 2 h (lane 4). Changes in IκBα were then measured by Western blot with GAPDH provided as a loading control. (**F**) Confluent HUVECs (Left) or MECs (Right) treated with TNFα and AXT107 as in (**A**–**D**) above and stained for TNFR1 or TNFR2. GAPDH is provided as a loading control.

To investigate the effects of AXT107 on TNFα-mediated EC inflammation, we cultured two EC cell lines for increasing amounts of time in the presence and absence of AXT107. Microvascular endothelial cells (MECs) were used to better simulate the smaller vessels found in the eye while human umbilical vein endothelial cells (HUVECs) were used for their ease of transfection. As NF-κB is the primary mediator of TNFα signaling, we focused our investigation on this pathway. Unsurprisingly, Western blots of HUVECs treated with 10 ng/mL TNFα showed a clear reduction in IκBα levels consistent with an inflammatory response as early as 30 min, and these reduced levels persisted for 6 h, the last timepoint tested (Figure 1A). By contrast, HUVECs pretreated with AXT107 showed a decrease similar to the untreated cells at 30 min, but IκBα levels were completely restored by the later time points (Figure 1A,B). A similar response was observed in MECs, except that IκBα levels at 30 min had already recovered in AXT107-treated cells to the same level observed in MECs that were not treated with TNFα (Figure 1D lane 5 vs. lane 1). For most subsequent experiments, we focused on 30 min and 4 h as these timepoints would sufficiently cover the initial drop and recovery of IκBα by AXT107, as well as allow for the translation of NF-κB response genes.

To further investigate the functional consequences of these changes, we assessed the levels of VCAM-1 and ICAM-1, which are both genes that are upregulated by the NF-κB transcription factor and are important adhesion molecules in inflammatory responses. A strong induction of both proteins was observed by two to four hours in DMSO-treated control samples from both HUVECs (Figure 1A, lanes 4 and 5 for VCAM-1 and Figure 1C lane 3 for ICAM-1) and MECs (Figure 1D, lanes 3 for both VCAM-1 and ICAM-1) while only lower levels were observed in cells pretreated with AXT107 (Figure 1B, lanes 4 and 5 and Figure 1D, lane 6 for VCAM-1 and Figure 1C lane 6 and Figure 1D lane 6 for ICAM-1).

The observation that IκBα levels were reduced in both control and AXT107-treated HUVECs at 30 min suggested that the peptide does not directly inhibit the initial activation of TNFα-mediated signaling but, instead, shortens the duration of the signaling and increases the rate of its resolution. Therefore, we tested if AXT107 could resolve inflammatory responses in HUVECs already exposed to TNFα. Cells were treated with TNFα for one hour followed by treatment with DMSO or AXT107 for 30 min or 2 h. IκBα levels were markedly reduced at both time points relative to untreated samples when DMSO was added following TNFα treatment (Figure 1E, lanes 1 vs. lanes 2 and 3) but were increasing (Figure 1E lane 2 vs. lane 5) or comparable to the IκBα levels in untreated or samples in which AXT107 treatment preceded TNFα addition for cells treated with AXT107 for 2 h after TNFα-pretreatment (Figure 1E lane 4 vs. lane 6).

Some anti-inflammatory agents have been reported to inhibit TNFα-mediated signaling by reducing the levels of the TNFRs [30]. Therefore, we investigated the effects of AXT107 and TNFα on the levels of TNFR1 and TNFR2 in both HUVECs (Figure 1F, left) and MECs (Figure 1F, right). Treatment with TNFα showed a slight reduction in TNFR1 levels for both cell types pre-treated with DMSO at 30 min (Figure 1F, lanes 1 and 7 vs. lanes 2 and 8) but recovered by 4 h (Figure 1F, lanes 3 and 9). Treatment with AXT107 appeared to reduce TNFR1 levels by itself in MECs (Figure 1F, lane 7 vs. lane 10) but was observed for both HUVECs and MECs when cells were treated with both AXT107 and TNFα at 30 min and 4 h (Figure 1F, lanes 1 and 7 vs. lanes 5, 6, 11, and 12). TNFR2 levels were weaker in MECs relative to HUVECs but appeared to remain unchanged in both cell lines regardless of treatment (Figure 1F).

**Figure 2:**
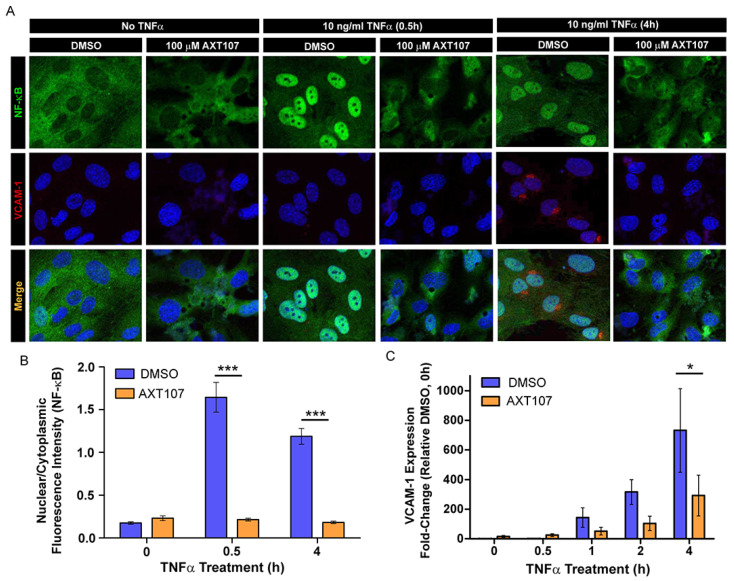
AXT107 regulates the intracellular localization and activity of nuclear factor kappa-light-chain-enhancer of activated B cells (NF-κB). (**A**) Representative immunofluorescence images of NF-κB (green), VCAM-1 (red), and 4′,6-diamidino-2-phenylindole (DAPI) (blue; VCAM-1 and merge only) staining in confluent HUVECs pre-treated with DMSO or 100 μM AXT107 followed by 10 ng/mL TNFα for 0, 0.5, or 4 h as indicated. (**B**) Image analysis of NF-κB fluorescence data showing the ratio of nuclear to cytoplasmic staining for HUVECs treated with DMSO (blue) or AXT107 (orange) following exposure to TNFα for varying amounts of time. *n* = 3, *** indicates significant difference between DMSO and AXT107-treated samples by two-way ANOVA, *p* < 0.001; df = 1; F = 137.8. (**C**) Reverse transcription quantitative polymerase chain reaction (RT-qPCR) analysis of VCAM-1 expression from HUVECs pretreated with DMSO (blue) or 100 μM AXT107 (orange) followed by 10 ng/mL TNFα for the indicated amounts of time. Data presented as fold-change relative to the DMSO-treated sample without TNFα treatment (0 h). *n* = 3, * indicates a significant difference between DMSO and AXT107-treated samples by two-way ANOVA, *p* < 0.05; df = 1; F = 5.092.

To correlate the changes in IκBα signaling to NF-κB activity, we investigated the intracellular distribution of NF-κB using fluorescence microscopy following exposure to TNFα (Figure 2A) and quantified the ratio of nuclear to cytoplasmic NF-κB by image analysis (Figure 2B). NF-κB in HUVECs treated with DMSO vehicle was found predominantly in the nucleus as early as 30 min after TNFα administration and remained in the nucleus for at least 4 h (Figure 2A columns 3 and 5, and Figure 2B). By contrast, AXT107-treated cells showed clear cytoplasmic staining at all durations of TNFα exposure tested (Figure 2A columns 2, 4, and 6, and Figure 2B). We also stained for the NF-κB-regulated protein VCAM-1 as an indicator of functional NF-κB. DMSO-treated cells showed perinuclear staining starting at 4 h after TNFα exposure (Figure 2A column 5). No VCAM-1 perinuclear staining, however, was observed in the AXT107-treated cells (Figure 2A column 6). As these changes in VCAM-1 are more directly related to NF-κB-induced gene expression, we investigated the relative effects of TNFα and AXT107 treatment on mRNA levels by reverse transcription quantitative polymerase chain reaction (RT-qPCR) (Figure 2C). HUVECs exposed to TNFα showed a clear induction of VCAM-1 mRNA by 4 h in DMSO-treated cells. However, treatment with AXT107 decreased VCAM-1 gene expression by 61% at 1 h, 69% at 2 h, and 60% at 4 h relative to the corresponding control. Together, these data indicate that AXT107 treatment reduces the duration of NF-κB localization to the nucleus following TNFα-mediated signaling and reduces the levels of inflammation-associated proteins by disrupting the induction of downstream genes typically induced by NF-κB.

### 2.2. AXT107 Reduces the Surface Expression of Inflammation-Induced Adhesion Molecules in Activated ECs and Inhibits Retinal Leukostasis

**Figure 3:**
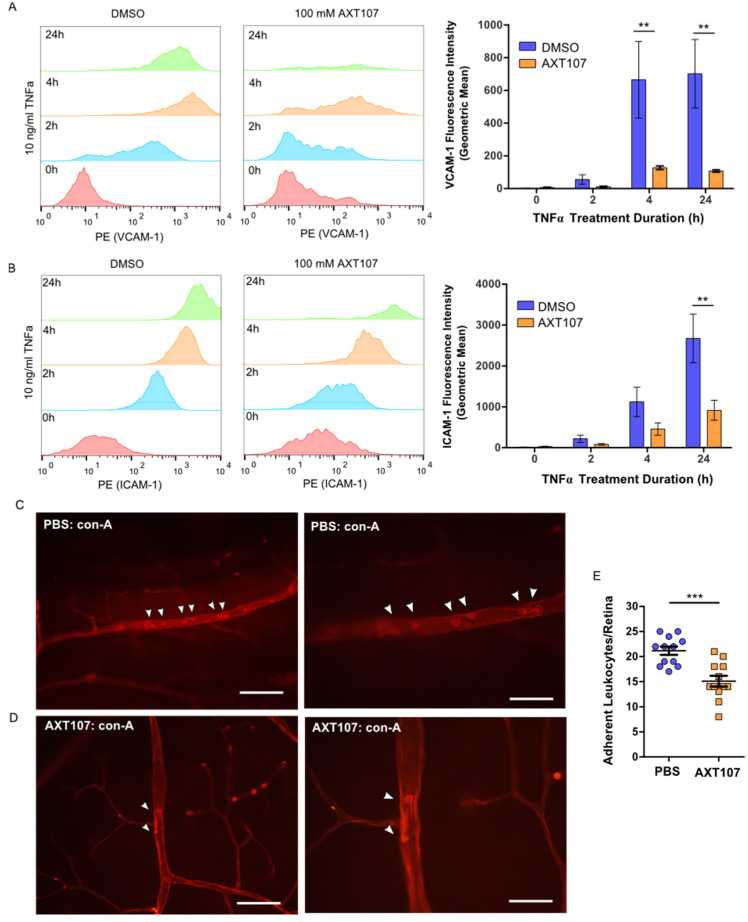
AXT107 inhibits TNFα-mediated upregulation of leukocyte adhesion molecules and leukostasis in eye vessels. (**A**,**B**) *Left—*Histograms for HUVECs pre-treated with DMSO or 100 μM AXT107 followed by 10 ng/mL TNFα for varying durations as indicated that were stained with PE-labeled antibodies for VCAM-1 (**A**) or ICAM-1 (**B**) in absence of permeabilization. *Right—*Quantification of the number of VCAM-1 (**A**) or ICAM-1 (**B**) molecules per cell determined by comparing sample geometric means relative to the DMSO sample at 0 h. *n* = 4 (3 at 24 h), two-way ANOVA with Bonferroni posttest, ** indicates significance relative to the corresponding DMSO control, *p* < 0.05, 0.01, and 0.001, respectively; df = 1; F = 14.88 (VCAM-1) or F = 11.32 (ICAM-1). (**C**,**D**) Representative immunofluorescence images of vessels in retina flat mounts from mouse eyes pre-treated with intraocular injections of PBS (**C**) or 1 μg AXT107 (**D**) followed by 50 ng TNFα that were flushed with PBS and stained for adherent leukocytes with fluorescein isothiocyanate (FITC)-conjugated conconavalin A. Higher-magnification images are shown on the right. Scale bars are 200 μm (left) and 100 μm (right). © Quantification of total leukocytes adherent in the blood vessels of isolated mouse retinas. *n* = 12, *** indicate significance relative to PBS control by Student’s *t*-test (two-tailed), *p* < 0.001.

In response to inflammatory signals, ECs alter their transcription profiles and enter an activated state. Activated ECs in blood vessels upregulate the expression of several luminal adhesion molecules, such as VCAM-1 and ICAM-1, in order to recruit leukocytes from circulation to inflamed sites. Our Western blot, RT-qPCR, and immunofluorescence data demonstrate a decrease in VCAM-1 and ICAM-1 levels in cells following AXT107 treatment. To confirm that these effects corresponded to changes in functional VCAM-1 and ICAM-1, we investigated the effects of AXT107 on surface VCAM-1 (Figure 3A) and ICAM-1 (Figure 3B) levels using flow cytometry. No permeabilization buffers were used and cells were kept on ice to prevent internalization, limiting the detection to only surface targets.

VCAM-1 levels for both control and AXT107-treated cells increased slightly after the first 2 h of TNFα exposure and then reached an apparent maximum by 4 h, where it remained for at least an additional 20 h. ICAM-1 levels responded similarly to TNFα treatment except that the expression appeared to increase over the entire 24 h period. However, AXT107-treated cells after TNFα exposure showed consistently lower surface levels of VCAM-1 and ICAM-1 than controls, with significant reductions of 81% and 85% observed at 4 and 24 h for VCAM-1 (Figure 3A) and 66% for ICAM-1 at 24 h (Figure 3B).

Exposure to inflammatory signaling molecules and growth factors can lead to an accumulation of leukocytes within the retinal vessels in a process known as retinal leukostasis. In severe cases, the accumulation of leukocytes in capillaries can plug these vessels and produce regions of nonperfusion, breakdown of the blood–retinal barrier, and tissue damage [31,32]. The expression of inflammatory adhesion molecules, such as VCAM-1 and ICAM-1, has been associated with this response [33,34]. Therefore, we investigated the effects of AXT107 treatment on leukostasis in the retinal vessels of mice following intraocular TNFα injection. Following AXT107 and TNFα treatment, mice were perfused with PBS to remove non-adherent cells and the vessels and leukocytes stained with fluorescein isothiocyanate (FITC)-conjugated concanavalin A (Figure 3C). Eyes treated with an intraocular injection of PBS vehicle contained 21.2 adherent leukocytes per retina on average following 24 h of TNFα treatment (Figure 3D). By comparison, AXT107-treatment significantly reduced the number of adherent leukocytes in retinal vessels by 29% to 15.1 cells per retina (Figure 3D).

### 2.3. TNFα Stimulates Tie2 Phosphorylation in AXT107-Treated ECs

**Figure 4:**
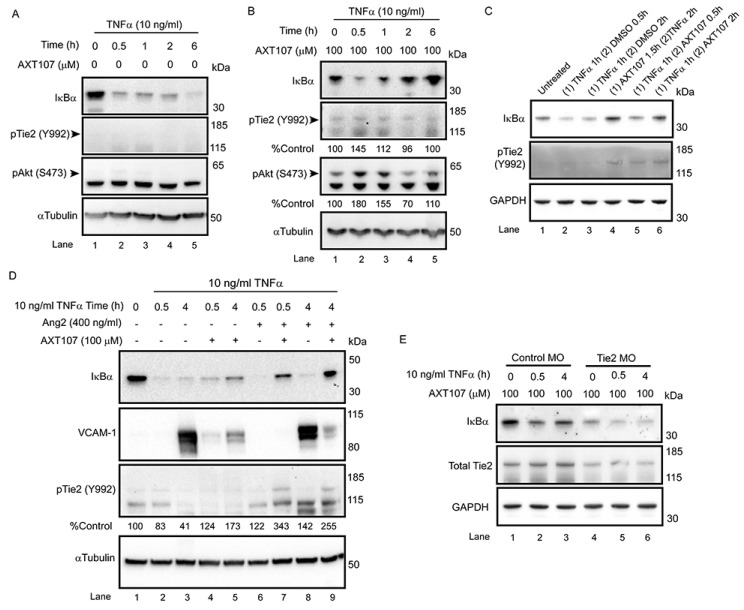
Tyrosine kinase with immunoglobulin and EGF-like domains (Tie2) phosphorylation contributes to the anti-inflammatory activity of AXT107. (**A**,**B**) Confluent HUVECs pre-treated with 100 µM AXT107 (**B**) or equivalent DMSO vehicle (**A**) as indicated for 90 min, followed by varying durations of 10 ng/mL TNFα, and analyzed by Western blot. IκBα and αTubulin data are the same as presented in Figure 1A,B as a reference for inflammatory status but now include the addition of phospho-Akt (pAkt) (S473) and phospho-Tie2 (pTie2) (Y992). Given the weak staining of some pTie2 bands, densitometry analysis was performed for pTie2 and pAkt images with AXT107-treated samples, and the loading control-normalized results are presented below the corresponding image as percentages of the value from the untreated control (lane 1). (**C**) Data from Figure 1E now including staining pTie2 (Y992). IκBα data are retained as a reference for inflammatory status. (**D**) Western blots of confluent HUVECs pre-treated with 100 µM AXT107 or equivalent DMSO vehicle followed by 400 ng/mL angiopoietin 2 (Ang2) 15 min and/or 10 ng/mL TNFα for various times as indicated and stained for IκBα, VCAM-1, and pTie2 with αTubulin as a loading control. Densitometry analysis of loading control-normalized pTie2 band intensity is indicated below the image as a percentage of the untreated control (lane 1). (**E**) Confluent HUVECs were treated with a control or Tie2 translation-blocking morpholino for 48 h followed by pre-treatment of AXT107 for 90 min and varying durations of treatment with 10 ng/mL TNFα. Samples were then stained by Western blot for IκBα and Tie2 with GAPDH as a loading control.

Activated Tie2 can regulate inflammation by interacting with ABIN2, which is presumed to inhibit NF-κB responses by blocking the activities of inhibitor of nuclear factor-κB kinase β (IKKβ). However, TNFα also stimulates the rapid release of Ang2 from EC Weibel–Palade bodies [35], which would be expected to dampen Tie2 signaling and potentiate inflammatory responses consistent with reports showing synergy between TNFα-signaling and Ang2 [24]. We have previously reported that AXT107 reorganizes Tie2 into clusters at EC–EC junctions that can be activated if exposed to either Ang1 or Ang2 [29], suggesting that the peptide’s anti-inflammatory properties may be connected to the activation of Tie2. In support of this, Tie2 phosphorylation was not observed for DMSO-treated HUVEC samples exposed to TNFα for 0 to 6 h but was observed in AXT107-treated samples at all time points (Figure 4A,B). Based on our previous work in which we observed slight variations in Tie2 phosphorylation when cells were treated with AXT107 alone [29], we suspect that the increase seen at the 0 h TNFα time point is the result of AXT107 in combination with basal Ang2 secretion and not an activity of the peptide alone. As shown through densitometric analysis, treatment with TNFα, which stimulates Ang2 release, further increased Tie2 phosphorylation, reaching a maximum level after 30 min. Elevated Tie2 phosphorylation was also observed in samples pre-treated with TNFα for 1 h followed by the addition of AXT107 (Figure 4C, lanes 1–3 vs. lanes 4–6). Activation of Tie2 by AXT107 is also associated with junctional localization, which specifically activates the downstream pathways associated with Akt. Consistent with previous reports, Akt phosphorylation was also increased and followed a pattern that matched Tie2 phosphorylation (Figure 4A,B).

The data strongly suggest that TNFα in combination with AXT107 can induce Tie2 phosphorylation and downstream signaling. Additionally, the requirement of Ang2 release and subsequent activation of Tie2 may provide a mechanistic explanation for the delay in IκBα recovery observed for AXT107-treated samples. To determine if the activation of Tie2 can directly influence the NF-κB signaling, we pretreated HUVEC cells with Ang2 in the presence and absence of AXT107 prior to exposure to TNFα. As seen previously, IκBα levels decreased by 30 min in cells treated with DMSO or AXT107 alone but recovered by 4 h in AXT107-treated cells (Figure 4D, lanes 2 vs. 4 and lanes 3 vs. 5). Interestingly, while the addition of Ang2 did not change the results for DMSO-treated samples, pretreatment of cells with Ang2 and AXT107 resulted in higher levels of IκBα even at the 30 min timepoint of TNFα-treatment (Figure 4D, lanes 6 vs. 7 and lanes 8 vs. 9). VCAM-1 levels followed the expected trends for the observed IκBα levels and were inversely correlated with IκBα levels, indicating that the effects on IκBα correlated with the activity of NF-κB. Moreover, strong phosphorylation of Tie2 was observed in the Ang2 and AXT107 combination samples, suggesting that the observed effect on IκBα protein levels was the result of Tie2 activation (Figure 4D, lanes 6 and 8 vs. lanes 7 and 9).

To confirm the importance of Tie2 in this model, we analyzed IκBα recovery in HUVEC cells following the knockdown of Tie2 translation. As siRNA against Tie2 did not provide sustained Tie2 reduction following the addition of TNFα, we decided to use morpholino (MO) technology. HUVECs treated with control MOs maintained consistent basal levels of Tie2 (Figure 4E, lanes 1–3) and demonstrated the expected recovery of IκBα levels after 4 h of TNFα in the presence of AXT107 (Figure 4E, lane 2 vs. 3). HUVECs treated with a translation-blocking MO against Tie2 showed a reduction in the levels of the protein, providing a model system to investigate the importance of Tie2 in IκBα regulation. Similar to the control MO samples, IκBα levels in samples with Tie2 MO decreased following 30 min of TNFα treatment (Figure 4E, lanes 4 vs. 5). However, the levels of IκBα did not recover at 4 h in the Tie2 MO sample as it did in the control MO sample (Figure 4E, lanes 3 vs. 6), providing further evidence that Tie2 is involved in the AXT107-mediated recovery from inflammation.

### 2.4. AXT107 Inhibits TNFα-Mediated Vascular Permeability and Instability

**Figure 5:**
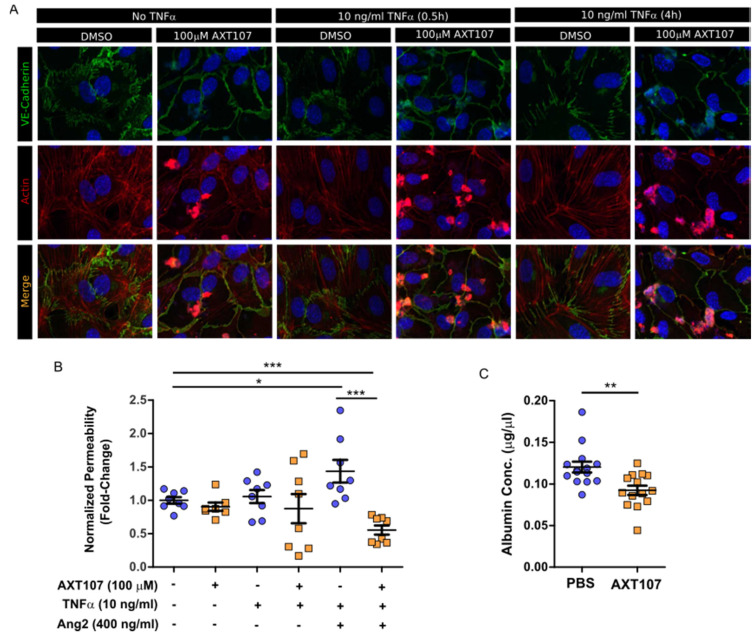
AXT107 inhibits TNFα-mediated endothelial cell (EC) junction instability and vascular leakage. (**A**) Representative images of confluent HUVECs pre-treated with DMSO or 100 μM AXT107 followed by 10 ng/mL TNFα for 0, 0.5, or 4 h, as indicated and stained for VE-cadherin (green), F-actin (red), or DAPI (blue). (**B**) Arbitrary fluorescence values for the diffusion of FITC-labeled dextran after 3 h across a HUVEC monolayer treated with 100 μM AXT107, DMSO, 10 ng/mL TNFα, and/or 400 ng/mL Ang2 as indicated and normalized to the untreated data. *n* = 8 (7 in AXT107 alone due to statistical outlier), * and *** indicate significance between indicated pairs, Mann–Whitney test, *p* < 0.05 and 0.001, respectively. (**C**) Albumin concentration collected from the vitreous of mouse eyes that were pre-treated with intraocular injections of PBS or 1 μg AXT107 followed by 50 ng TNFα and measured by ELISa. *n* = 14, ** indicates significance, Mann–Whitney test, *p* < 0.01.

As part of the inflammatory response, gaps begin to form at EC junctions to allow the leakage of plasma proteins into the interstitial tissue in order to support incoming leukocytes [14]. If unresolved, the vascular hyperpermeability can contribute to disease progression. Specifically, macular edema caused by vascular leakage is the leading cause of vision loss in uveitis [36]. AXT107 has been previously shown to stabilize EC junctions by potentiating Tie2-activation by Ang2 [29]. To determine if this stabilization would be maintained under inflammatory conditions, cultures of confluent HUVECs were treated with either DMSO or AXT107 followed by exposure to TNFα for 30 min or 4 h. Changes in junctions were then monitored by immunofluorescent staining of VE-cadherin and F-actin (Figure 5A). VE-cadherin staining in DMSO control cells (Figure 5A, columns 1, 3, and 5) was jagged in appearance and associated with F-actin that crossed radially through the core of the cell, an arrangement consistent with lower junctional stability. Conversely, pretreatment of the cells with AXT107 (Figure 5A, columns 2, 4, and 6) resulted in a smoother VE-cadherin appearance and peripheral F-actin at all time points, an arrangement associated with more stable junctions. We then investigated if this apparent increase in junctional stability influenced the permeability of EC monolayers using a Transwell culture system and FITC-labeled dextran (Figure 5B). In this model, HUVECs were cultured to confluency on a semi-permeable membrane between the two chambers and the permeability was determined by the diffusion of the FITC-labeled dextran from the lower to the upper chamber. AXT107 treatment alone had no significant effect on permeability relative to DMSO controls. Likewise, TNFα-treatment did not significantly affect monolayer permeability in cells treated with DMSO or AXT107 despite higher variability in the data. When TNFα was present in combination with Ang2, however, the diffusion of dextran through the EC monolayer significantly increased relative to vehicle control cells. Interestingly, treatment with AXT107 not only prevented this increase in diffusion but also reduced the permeability of the monolayer significantly below that of the vehicle control.

To determine if these effects would recapitulate in vivo, we administered AXT107 or PBS vehicle by intraocular injection, followed by TNFα 24 h later, and quantified the amount of serum albumin that had leaked into the vitreous by ELISA after another 24 h (Figure 5C). Vitreous from eyes pretreated with AXT107 showed a significant 20% reduction in serum albumin concentration compared to PBS controls. Together, these data provide evidence that AXT107 inhibits inflammation-induced vascular leakage.

## 3. Discussion

Chronic inflammation is a major contributor to morbidity in many ocular diseases, such as DME, DR, RVO, AMD, uveitis, and DED, and, if left untreated, can cause debilitating tissue damage. Failure to treat most of these conditions can lead to blindness, while people with untreated DED experience pain and discomfort. Although the standard of care for patients with ocular diseases involving VEGF-induced vascular leakage and neovascularization, such as DME and wet AMD, is treatment with anti-VEGF therapies, fewer than 50% of these patients achieve a ≥15 letter improvement in vision. As inflammation is an underlying condition in these diseases [4,37,38], using an agent that inhibits VEGF, activates Tie2, and helps resolve underlying inflammation may improve outcomes for these patients. The work reported here shows that AXT107, a 20-mer peptide that inhibits VEGF signaling and activates Tie2 though endogenously released Ang2 to help resolve underlying inflammation, could be used to treat DME, DR, RVO, AMD, uveitis, DED, and other ocular diseases that are inadequately treated with the current standard of care therapies.

**Figure 6:**
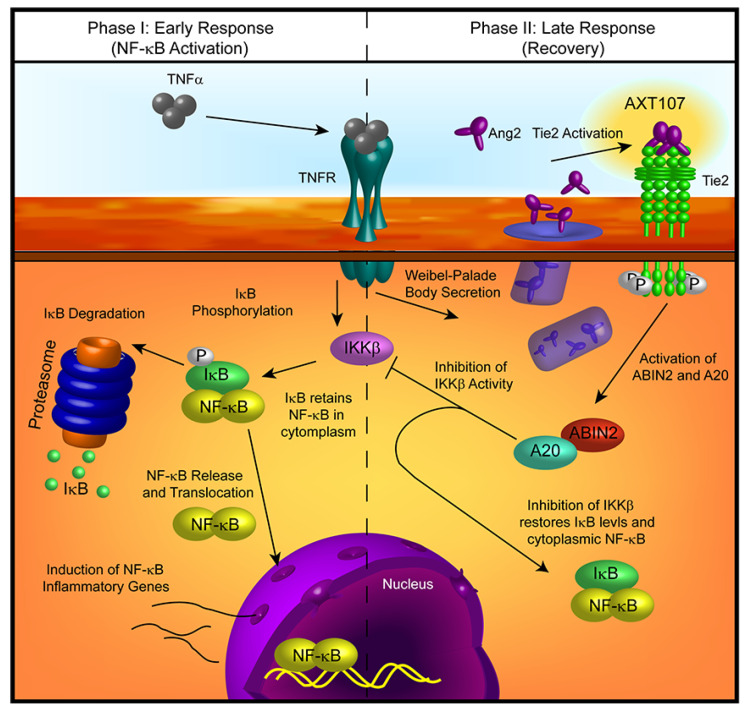
Model of AXT107-mediated regulation of TNFα signaling in endothelial cells. *Early phase* (left)—Initial activation of TNFR1 by TNFα is unaffected by AXT107 treatment, leading to the activation of downstream targets, including inhibitor of nuclear factor-κB kinase β (IKKβ). IKKβ phosphorylates IκB, resulting in its separation from NF-κB and degradation at the proteasome. NF-κB is then free to enter the nucleus and activate inflammatory response genes. *Late phase* (right)—Stimulation of TNFR1 induces the rapid release of Ang2 from Weibel–Palade bodies, which, in combination with AXT107, stimulates Tie2 autophosphorylation. Phosphorylated Tie2 then activates the downstream factors A20 binding inhibitor of NF-κB 2 (ABIN2) and A20, which inhibit IKKβ activity and the consequential loss of IκB. The restoration of IκB levels reverses NF-κB nuclear localization and attenuates the TNFα-mediated inflammatory response.

Treatment with AXT107 does not directly inhibit TNFα-mediated signaling, as both the DMSO and peptide-treated samples showed a clear reduction in IκBα levels following stimulation of TNFα in the earliest time points, particularly in HUVECs. Moreover, it does not appear that AXT107 treatment alone can initiate the recovery of IκBα but, instead, AXT107 stimulates its recovery following the addition of TNFα. As a possible explanation for this delayed effect, we propose that the anti-inflammatory activities of AXT107 are mediated partly through the Tie2 signaling pathway. In quiescent vessels, Tie2 signaling is usually activated through ligation to Ang1 supplied by perivascular cells while inflammatory signals, including TNFα, stimulate the rapid secretion of Ang2 from Weibel–Palade bodies stored in ECs, which bind and inhibit Tie2 activation. However, AXT107 has been previously shown to reorganize Tie2 within the cell so that it may be stimulated by Ang2 as well. Therefore, we propose a model where AXT107 does not block the initial steps of TNFα inflammation (Figure 6, left side), which results in the initial drop of IκBα observed at 30 min, but utilizes the released Ang2 to shut down IκBα degradation and the downstream NF-κB inflammatory responses through a Tie2-mediated pathway (Figure 6, right side). In support of this model, phosphorylation of Tie2 and the downstream effector Akt was observed in samples treated with both AXT107 and TNFα regardless of order of administration (Figure 4C) and, in the case of AXT107 pretreatment, the phosphorylation levels appeared to be inversely correlated to the reduction in IκBα. Furthermore, the initial drop of IκBα at 30 min could be avoided by pretreatment with AXT107 and Ang2 (Figure 4D) before the addition of TNFα, presumably through the activation of Tie2-associated anti-inflammatory signaling before the initiation of TNFα-mediated responses, or sustained despite AXT107 addition through the knockdown of Tie2 (Figure 4E).

While both HUVECs and MECs clearly demonstrated a reduction in inflammatory responses to TNFα exposure following treatment with AXT107, peptide-treated MECs did not show the same initial drop in IκBα at the 30 min timepoint. At this point, we cannot rule out the possibility that another mechanism entirely is responsible for this difference between cell types; however, it is also possible that differences between the cell types allow for faster recovery by the MECs using the same mechanism. For instance, TNFR1 levels in MECs may be more sensitive to AXT107-induced degradation, resulting in a reduced effect in the downstream effects on IκBα levels compared to HUVECs. Notably, a clear drop in TNFR1 can be observed in MECs exposed to AXT107 alone (Figure 1F, lane 7 vs. lane 10), suggesting that the TNFR1 levels are reduced during the peptide pre-treatment before TNFα is introduced. Alternatively, while our previous work clearly shows that AXT107 can potentiate Tie2 activation by exogenous Ang2 in both cell types [29], most of the experiments presented here rely on endogenously produced Ang2. As such, differences in the expression of Ang2 or any of the downstream components essential for Tie2-mediated anti-inflammatory signaling (e.g., Tie2, ABIN2, etc.) could also account for cell type differences. For instance, microvascular cells show a higher fold increase in Tie2 expression in response to hypoxia or inflammatory factors compared to HUVECs [39]. As such, it may be that other factors, like the serum starvation used in this work, could induce more Tie2 expression in MECs compared to HUVECs. Finally, the expression of some integrins differ between microvascular and macrovascular cells and, as the targets of AXT107, may alter their responses to the peptide [40].

Conditions in which AXT107 is present before the addition of TNFα to ECs simulate a prophylactic use of AXT107. In this manner, AXT107 could be useful for preventing the incidental inflammation resulting from intraocular injections when the peptide is used to treat other diseases, such as DME and AMD. However, for the effective treatment of inflammatory disease, it is important to have evidence that AXT107 can inhibit pre-existing inflammation. We found that the anti-TNFα effects of AXT107 are the same whether AXT107 is added before or after the addition of TNFα to ECs (Figure 1E lane 4 vs. lane 6), and the levels of IκBα in either case were comparable to those in cells that were not treated with TNFα (Figure 1E, lane 1) and higher than those in cells treated with TNFα when AXT107 was absent (Figure 1E, lane 3). Higher levels of IκBα sequester NF-κB in the cytoplasm, hence shutting off the synthesis of pro-inflammatory molecules and thus inhibiting inflammation. These data show that AXT107 can be used to treat pre-existing ocular disease with underlying inflammation.

AXT107 reduces the level of TNFR1 but does not affect the level of TNFR2 (Figure 1C,D, lanes 1 vs. lanes 5 and 6). These lower levels of TNFR1 may dampen the sustained signaling by TNFα and could partially explain the 30 min delay in IκBα recovery observed in AXT107-treated cells. Some of the side effects of anti-TNFα agents are thought to result from their inhibition of both TNFR1 and TNFR2 because they work by binding TNFα itself and preventing it from binding to both receptors. Unlike TNFR1, TNFR2 activity also stimulates neuroprotective and vascular stabilizing functions [41,42]. These properties suggest that agents that selectively inhibit TNFR1 may be just as effective as current anti-TNFα agents but may have fewer side effects because they do not suppress the beneficial effects of TNFR2 activation [10]. Thus, the AXT107-induced selective degradation of TNFR1 could be advantageous. Notably, TNFR2 has been linked with the activation of Akt [41], which is also upregulated by AXT107 through the stimulation of Tie2 signaling [29]. Therefore, treatment with AXT107 appears to downregulate the negative aspects of TNFα-signaling without negating the positive neural and vascular trophic effects.

Retinal leukostasis occurs when ECs in capillaries that are activated at sites of inflammation increase surface expression of VCAM-1 and ICAM-1, causing adhesion of leukocytes [33,34]. The adhered leukocytes block blood flow, which causes nonperfusion and ischemia, leading to tissue damage and breakdown of the blood–retinal barrier (BRB), which can occur in posterior uveitis [31,32]. The degradation of the BRB is characterized by the loss of junction stabilizing molecules, including zona occludens, occludins, and VE-cadherins, and a consequential increase in vascular permeability. This can result in macular edema, a condition that is a main cause of vision loss in uveitis [43]. AXT107 was able to restore the integrity of EC junctions and reduce vascular permeability both in vitro and in vivo (Figure 5A–C). Moreover, AXT107 potently inhibits TNFα-induced expression of both ICAM-1 and VCAM-1 (Figure 1, Figure 2 and Figure 3) and leukostasis (Figure 3E), as shown by the reduced number of leukocytes adhered to the capillaries compared to leukostasis in control animals. Similarly, corticosteroids have been shown to inhibit diabetes-induced leukostasis in animal models [44]. The data we report here together with the finding that anti-inflammatory corticosteroids inhibit leukostasis in diabetes models suggest that AXT107 could also be used to inhibit leukostasis caused by both diabetes and uveitis.

Currently, the agents used to treat underlying inflammation of ocular diseases include anti-inflammatory agents such as corticosteroids, systemic anti-TNFα agents such as adalimumab (Humira^®^), and immunosuppressives (e.g., cyclosporine A), which can cause cataracts, glaucoma, susceptibility to infections and cancer, and other side effects. AXT107 could be superior to these other agents because of its unique mechanism of action. We have previously shown that AXT107 inhibits neovascularization and vascular leakage by simultaneously inhibiting VEGF signaling and activating Tie2 by converting the ligand Ang2 from an antagonist of Tie2 to an activator of Tie2 [29]. Here, we show that AXT107 resolves existing inflammation by utilizing the same mechanism of Ang2-dependent activation of Tie2. This is particularly important as, in addition to its role in promoting vascular leakage by inhibiting Tie2 phosphorylation, Ang2 also promotes inflammation by enhancing the effects of TNFα. We have previously shown that Ang2 overexpression alone was sufficient to increase leakage in retinal vessels [28] and, here, we demonstrate that Ang2 can enhance TNFα-mediated permeability relative to TNFα alone (Figure 5B). These data are consistent with the observations that the release of Ang2 stored within ECs by TNFα sensitizes ECs to TNFα stimulation [24]. Moreover, as the canonical agonistic ligand of Tie2, Ang1, is still able to function in the presence of AXT107, treatment with the peptide would not disrupt normal stabilizing and anti-inflammatory signals by Ang1 following the resolution of any disease conditions. As such, AXT107 can attenuate both TNFα signaling directly, like other currently used anti-inflammatory agents, as well as disrupt the synergy between Ang2 and TNFα in addition to its already potent effects on pathological neovascularization and vascular leakage.

The data we report here suggest that AXT107 could also be used to treat uveitis, especially posterior uveitis, which is an inflammation of the retina and choroid. Uveitis, the inflammation of the uvea consisting of the iris, choroid, and ciliary body, is the third leading cause of blindness in the US [45]. It accounts for approximately 30,000 new cases of legal blindness in the US and for 10–15% of all cases of total blindness [46]. Non-infectious uveitis is a collection of autoimmune diseases that are classified as idiopathic [47,48] or that occur as part of systemic autoimmune diseases such as ankylosing spondylitis, reactive arthritis, enteropathic arthropathy or psoriatic arthritis, Behçet’s disease, sarcoidosis, systemic lupus, and multiple sclerosis [46,47,49], and involve inflammatory cytokines [43]. The most common therapies for treating uveitis are general anti-inflammatory drugs like corticosteroids, immunosuppressives such as cyclosporine-A, methotrexate, and mycophenolates, and anti-TNFα agents, such as Humira [50]. However, the serious side-effects associated with several of these therapies (as described above) emphasize an opportunity for improvement. As such, the alternative mechanism of AXT107 may provide relief from inflammation without the associated negative responses of other agents.

AXT107 has a unique mechanism in that it inhibits VEGF signaling, activates Tie2, and helps resolve the underlying inflammation of many ocular vascular diseases. Furthermore, these anti-inflammatory properties of AXT107 on ECs and in mouse models of inflammation provide support for potentially developing AXT107 to treat additional ocular diseases including uveitis and DED. We have also found that AXT107 can be safely administered intravitreally in rabbits and forms a gel upon injection that stays below the visual axis and releases efficacious peptide in a sustained manner for at least two months [28]. We suspect that greater doses of the peptide would form a larger gel that could maintain this sustained release and efficacy over an extended period. This combination of sustained delivery, vessel stabilization, anti-angiogenic, and anti-inflammatory properties in a single agent could provide a lasting, more complete treatment of ocular diseases with fewer injections than the current standards of care, dramatically reducing the treatment burden for patients.

## 4. Materials and Methods

### 4.1. Cell Culture and Peptide Handling

Human umbilical vein endothelial cells (HUVECs; Lonza, Walkersville, MD, USA) and microvascular endothelial cells (MECs; Lifeline Cell Technology, Frederick, MD) were maintained in VascuLife^TM^ VEGF endothelial media and VascuLife^TM^ VEGF-MV endothelial media (Lifeline Cell Technology), respectively, at 37 °C in 5% CO_2_ and used between passages 2 and 7. AXT107 was manufactured by solid-phase synthesis by New England Peptide (Gardner, MA, USA). For cell culture experiments, the lyophilized peptide was dissolved in 100% DMSO and diluted to 5% before addition to cultures. The total amount of DMSO never exceeded 0.25% in any experiment and additional DMSO vehicle was added where needed to normalize all samples. Culture dishes, 6- or 96-well plates, were coated with 10 μg/mL fibronectin 1 (FN1; Sigma-Aldrich, St. Louis, MO, USA) in PBS for 2 h at 37 °C. The FN1 solution was then removed and the cells cultured normally. All cells in an experiment were serum-starved two hours before other experimental factors were added to the longest time point tested, except for the 24 h time points, which were initiated separately from other samples to avoid excessive cell loss. For serum starvation, cells were washed twice with Dulbecco’s phosphate buffered saline (dPBS) containing calcium and magnesium, once with endothelial base media (Lifeline Cell Technology, Frederick, MD, USA), and cultured in the desired amount of endothelial base media. For in vivo studies, AXT107 was dissolved in sterile PBS.

### 4.2. Western Blotting

HUVEC or MEC cells were cultured to confluency in FN1-coated 6-well plates and serum-starved for 2 h as described above. For AXT107 pre-treated samples, 100 µM AXT107 or equivalent DMSO vehicle (0.25% in water) was added to cells at the same time as serum starvation. TNFα (10 ng/mL) addition was staggered so that all samples could be harvested at once. Ang2 (400 ng/mL) was added 15 min before TNFα addition where applicable. After treatments, cells were washed twice in cold DPBS with calcium and magnesium (Corning, Corning, NY, USA) and collected in 120 µL of 1× Blue Loading Buffer (Cell Signaling Technology, Danvers, MA, USA; Cat#: 56036). Samples were then boiled, sonicated, and stored at −20 °C until resolved by SDS-PAGE. Proteins were transferred to nitrocellulose or PVDF membranes and the contents analyzed by Western blot using primary antibodies listed in Table 1. Bands were imaged using the horseradish peroxidase (HRP)-conjugated anti-rabbit or anti-mouse secondary antibodies (Kindle Biosciences, Greenwich, CT, USA) and the KwikQuant imaging system (Kindle Biosciences). Where applicable, densitometry measurements were made using ImageJ software (v. 1.52p, National Institutes of Health, USA). The obtained values were only compared to bands within the same blot image.

For Tie2 knockdown experiments, HUVECs were grown to confluency as above and then transferred into MEC growth media for the higher fetal bovine serum (FBS) concentrations. The cells were then treated with 8 µM Endo-Porter PEG (Gene Tools, Philomath, OR, USA) followed by 15 µM of the prepared control oligo (PCO)-Standard Control-100-F or Tie2 translation-blocking (sequence CTGGCTAAAGAGTCCATGCCATGCTTCCCC) morpholinos (Gene Tools). The cultures were then incubated for 48 h followed by serum starvation and treatments as described for other Western blots above.

### 4.3. Immunofluorescence

Immunofluorescence was performed using modifications to our previously described procedures [29]. FN1-coated, glass-bottom 96-well plates were seeded with 1.2 × 10^4^ HUVECs in EC growth media and cultured overnight at 37 °C. After 24 h, the cells were washed once with dPBS containing Ca^2+^ and Mg^2+^, fresh growth media were added, and the cells were returned to the incubator for an additional 24 h. Cells were then washed twice with dPBS containing Ca^2+^ and Mg^2+^, and serum-starved for 30 min in EC base media. AXT107 (100 µM) or equivalent DMSO was then added and the plate incubated for another 90 min. TNFα (10 ng/mL) was then added for the durations indicated but staggered so that all wells were harvested simultaneously, at which point signaling was stopped by two washes in cold dPBS. The cells were then formalin fixed for 15 min in 10% neutral buffered formalin, washed three additional times in dPBS, and blocked for 1 h in blocking buffer (5% normal goat serum; 0.3% Triton X-100 in dPBS). Samples were then incubated overnight with antibody solutions against NF-κB, VCAM-1, or VE-cadherin (see Table 1) diluted 1:150 in blocking buffer. After three washes samples were then fluorescently labeled for 1 h with Alexa Fluor 488-conjugated anti-rabbit or Alexa Fluor 594-conjugated anti-mouse Fab2 secondary antibodies (Cell Signaling; Cat#: 4412 and 8890) diluted 1:300 in blocking buffer. If actin staining was required, wells were washed twice in dPBS followed by a 20 minute incubation in a solution of Alexa Fluor 555-conjugated phalloidin (Cell Signaling Technology; Cat#: 8953) prepared as described by the manufacturer. Following secondary antibody or actin staining, wells were again washed twice in dPBS and stained with 4′,6-diamidino-2-phenylindole (DAPI). The wells were then exchanged for dPBS for imaging using the BD Pathway 855 system (BD Biosciences, Franklin Lakes, NJ, USA) or the Zeiss AxioObserver with the LSM700 confocal module (Zeiss, Oberkochen, Germany). Analysis of NF-κB subcellular localization was conducted using ImageJ software (National Institutes of Health, USA) by determining the integrated fluorescence intensity of the NF-κB stain that overlapped with the DAPI stain, which corresponds to the nuclear contribution of the signal, and subtracting this from the total integrated fluorescence intensity to determine the cytoplasmic fraction. Data were presented as the ratio of nuclear to cytoplasmic fluorescence signals.

### 4.4. In Vitro Gene Expression

HUVEC cells were grown to confluency, serum-starved, and treated with AXT107, DMSO, and/or TNFα as described for Western blots above. RNA was then extracted using the RNeasy kit (Qiagen, Hilden, Germany) according to the manufacturer’s instructions. For lysis, 350 µL of RLT buffer was added to each well, collected by scraping, transferred to a QIAshredder (Qiagen), and centrifuged at 12,000× *g* before proceeding with the RNeasy protocol. cDNA was generated from 1 µg RNA using the High-Capacity cDNA Reverse Transcription Kit (Applied Biosystems Foster City, CA; Cat#: 4368814) according to the manufacturer’s instructions. PCR was performed on the Quantstudio 12K Flex system (Thermo Fisher Scientific, Waltham, MA, USA) using the TaqMan^TM^ Gene Expression Master Mix (Applied Biosystems, Foster City, CA, USA; Cat#: 4369016) with 0.7 µg of cDNA and the following TaqMan^TM^ (Applied Biosystems) 5(6)-carboxyfluoroscein (FAM)-labeled probes: VCAM-1 (Hs01003372_g1), glyceraldehye 3-phosphate dehydrogenase (GAPDH) (Hs02786624_g1), and Actb (Hs01060665_g1). Relative fold changes were determined by the ΔΔCT method using the GAPDH and Actb expression of the DMSO samples without TNFα as controls.

### 4.5. Flow Cytometry

HUVEC cells were cultured to confluency in FN1-coated 6-well plates. The cells were then serum-starved for 2 h with 100 µM AXT107 or equivalent DMSO added after 30 min. TNFα (10 ng/mL) was then administered for 0, 2, 4, or 24 h. The addition of TNFα was staggered so that all samples could be harvested at the same time. The cells were then washed twice with dPBS without Ca^2+^ and Mg^2+^, detached with 0.025% Trypsin solution (Corning), and neutralized with Trypsin Neutralizing Solution (Lifeline Cell Technology). Cells were then washed once in dPBS without Ca^2+^ and Mg^2+^, resuspended in stain buffer (PBS, 4% FBS, and 0.09% sodium azide), and transferred into flow tubes at 1 × 10^5^ cells per tube. The cells were then washed twice in stain buffer, decanted, and then stained with 5 µL of PE-conjugated primary antibodies for VCAM-1 and ICAM-1 added directly to the remaining volume (approximately 100 µL). After 40 min, the cells were washed three times with stain buffer. During these last washes, a tube of Quantibrite^TM^ PE Quantitation beads (BD Biosciences) was also prepared. Data were collected using the FACScalibur flow cytometer and FACS Diva software (BD Biosciences) and analyzed with FlowJo software (BD Biosciences). The number of molecules per cell was quantified from geometric means using the Quantibrite^TM^ bead standards (BD Biosciences) according to the manufacturer’s protocol.

### 4.6. FITC-Dextran Permeability Assay

Permeability assays were performed using modifications to our previously described procedures [29]. Transwell, 24-well inserts (Corning) were coated with 7.5 μg/cm^2^ FN1 for two hours at 37 °C, aspirated, and then dried for 30 min at room temperature. Top chambers were then seeded with 1 × 10^5^ HUVECs in 100 μL of VascuLife^TM^ VEGF endothelial media (Lifeline Cell Technologies) and incubated for 30 min at room temperature to avoid the accumulation of cells at the edge of the wells. The cells were then cultured for 24 h with an additional 200 µL and 1 mL of growth media in the top and bottom chambers, respectively. To improve the robustness of the assay, wells were seeded with an additional 1 × 10^5^ HUVECs and the media replaced. After another 24 h of culture the media was removed from top and bottom chambers and the cells gently washed two times with dPBS, once with endothelial basal media (Lifeline Cell Technologies), and serum starved for 2 h. The cells were then treated with 100 μM AXT107 or equivalent DMSO vehicle for 1.5 h followed by a 3 h incubation with 400 ng/mL Ang2, 10 ng/mL TNFα, and/or PBS control in the top chamber, and 25 μg/mL FITC-Dextran (40 kDa MW) in the bottom chamber. For detection, 10 μL samples were taken from the top chamber, diluted 1:10 in water, and the fluorescence quantified with a Biotek Synergy HT fluorescence plate reader (Biotek Instruments, Winooski, VT, USA).

### 4.7. Mouse Model of TNFα-Induced Inflammation and Leukostasis

Adult C57BL/6 mice received an intravitreal injection of 1 μL containing 1 μg of AXT107 in one eye or 1 μL of PBS in the fellow eye. Twenty-four hours later, both eyes were treated with 50 ng of recombinant mouse TNFα (BioLegend, San Diego, CA, USA) by intravitreal injections. After 24 h, mice were anesthetized with a mixture of ketamine and xylazine hydrochloride and then divided in two groups: (1) Vitreous samples from both eyes were collected to measure albumin levels using the albumin ELISA kit (ab108791, Abcam, Cambridge, MA, USA); (2) the total number of leukocytes adhering to the retinal vessels was counted. For the second group of animals, mice were first perfused with 5 mL of PBS to wash out non-adherent leukocytes from the blood vessels. Mice were then immediately perfused with fluorescein isothiocyanate (FITC)-conjugated Concanavalin A (20 μg/mL in 5 mL of PBS, pH 7.4; Vector Labs, Burlingame, CA, USA), as previously described [51], to label adherent leukocytes and vascular endothelial cells. After the eyes were removed and fixed in formalin, retinas were flat-mounted, prepared for quantification [52], and examined with the Axioskop microscope, and the images were digitized. The total numbers of leukocytes adhering to the retinal vessels were counted with the investigator being masked as to the nature of the specimen.

### 4.8. Statistical Methods

In experiments in which a single experimental group was compared to a single control group, data were checked for normality using the D’Agostino and Pearson omnibus normality test, and statistical comparisons were made using Student’s *t*-test if the data were normally distributed or the Mann–Whitney test if they were not. Group comparisons were made using two-way ANOVA with the Bonferroni post-test. All statistical methods are indicated in the corresponding figure legends and conducted using GraphPad Prism^®^ software (v. 5.0, GraphPad Software, Inc., San Diego, CA, USA). A *p*-value less than 0.05 was considered significant. Significance and sample size are reported in figure legends. All image analysis was performed with ImageJ. Experimental replicates correspond to separate animals for in vivo experiments, with one eye used for control and the other for treatment, and separate passages or frozen stocks for cell-based experiments with each replicate experiment separated by at least a week.

### 4.9. Study Approval

Mice were used in accordance with the Association for Vision Research and Ophthalmology Guidelines on the care and use of animals in research and approved by the Johns Hopkins University IACUC.

## Figures and Tables

**Table 1:** Antibodies used in this study.

Target	Application ^1^	Manufacturer	Cat Number
**GAPDH**	WB	Cell Signaling	2118
**ICAM-1**	FC (PE)	BioLegend	322707
**ICAM-1**	WB	Cell Signaling	4915
**IκBα**	WB	Cell Signaling	9242
**NF-κB (p65)**	IF	Cell Signaling	8242
**phospho-Akt (S473)**	WB	Cell Signaling	4058
**phospho-Tie2 (Y992)**	WB	Cell Signaling	4221
**Tie2**	WB	Cell Signaling	4224
**TNFR1**	WB	Cell Signaling	3736
**TNFR2**	WB	Cell Signaling	3727
**VCAM-1**	FC (PE)	R&D	FAB5649
**VCAM-1**	WB; IF	R&D	BBA5
**VE-Cadherin**	IF	Cell Signaling	2500
**αTubulin**	WB	Abcam	ab4074

^1^ WB—Western blot; IF—Immunofluorescence; FC—Flow Cytometry (label).
